# Sustained Release of Antibacterial Lipopeptides from Biodegradable Polymers against Oral Pathogens

**DOI:** 10.1371/journal.pone.0162537

**Published:** 2016-09-08

**Authors:** Lea H. Eckhard, Yael Houri-Haddad, Asaf Sol, Rotem Zeharia, Yechiel Shai, Shaul Beyth, Abraham J. Domb, Gilad Bachrach, Nurit Beyth

**Affiliations:** 1 Department of Prosthodontics, the Hebrew University–Faculty of Dental Medicine, Jerusalem, Israel; 2 Institute of Dental Science, the Hebrew University–Faculty of Dental Medicine, Jerusalem, Israel; 3 Department of Biological Chemistry, the Weizmann Institute of Science, Rehovot, Israel; 4 Orthopedic Surgery Department, Hadassah Medical Center, Jerusalem, Israel; 5 Institute for Drug Research, School of Pharmacology, Faculty of Medicine, the Hebrew University, Jerusalem, Israel; Helsingin Yliopisto, FINLAND

## Abstract

The development of antibacterial drugs to overcome various pathogenic species, which inhabit the oral cavity, faces several challenges, such as salivary flow and enzymatic activity that restrict dosage retention. Owing to their amphipathic nature, antimicrobial peptides (AMPs) serve as the first line of defense of the innate immune system. The ability to synthesize different types of AMPs enables exploitation of their advantages as alternatives to antibiotics. Sustained release of AMPs incorporated in biodegradable polymers can be advantageous in maintaining high levels of the peptides. In this study, four potent ultra-short lipopeptides, conjugated to an aliphatic acid chain (16C) were incorporated in two different biodegradable polymers: poly (lactic acid co castor oil) (PLACO) and ricinoleic acid-based poly (ester-anhydride) (P(SA-RA)) for sustained release. The lipopeptide and polymer formulations were tested for antibacterial activity during one week, by turbidometric measurements of bacterial outgrowth, anti-biofilm activity by live/dead staining, biocompatibility by hemolysis and XTT colorimetric assays, mode of action by fluorescence-activated cell sorting (FACS) and release profile by a fluorometric assay. The results show that an antibacterial and anti-biofilm effect, as well as membrane disruption, can be achieved by the use of a formulation of lipopeptide incorporated in biodegradable polymer.

## Introduction

The oral environment contains various microorganisms some of which are pathogenic species involved in dental caries, periodontal diseases and endodontic lesions [1]. These bacteria include *Streptococcus mutans*, *Actinomyces naeslundii*, *Porphyromonas gingivalis*, *Fusobacterium nucleatum* and *Enterococcus faecalis*. They differ one from the other but share the crucial ability to form dental biofilm, a dynamic, active metabolic structure that presents a challenge to antibiotics [2, 3].

Antimicrobial peptides (AMP's) are developmental components of the innate immune system that are produced by different types of organisms. AMPs are potent and efficient against various pathogens owing to their structure and charge [4]. Cationic AMPs were proposed for use as potential anti-infective compounds against antibiotic-resistant virulent strains of microorganisms [5]. In general, AMP activities include: the ability to kill a wide range of pathogens, as well as their potential to activate anti-inflammatory cells and recruit them to the injured tissue from the blood stream [6]. Although the complex mechanism of action of AMPs, is not fully clear, it is generally accepted that these peptides selectively disrupt cell membranes owing to their amphipathic structural arrangement [7]. However, microbes have evolved several resistance strategies to circumvent AMP function, such as alteration of cell net surface charge [8], intracellular AMP degradation [9] and AMP export via efflux pumps [10].

Another potent family of antibacterial factors is the native lipopeptides that are produced only by bacteria and fungi during their cultivation on various carbon sources. However, as native lipopeptides are not cell-selective, they are toxic to mammalian cells [11], although, the lipopeptide daptomycin which is active only against Gram-positive bacteria, was approved by the FDA for the treatment of skin infections caused by *Staphylococcus aureus* [12]. Most native lipopeptides consist of a short (six to seven amino acids) linear or cyclic peptide sequence, with a net positive or negative charge, to which a fatty acid moiety is covalently attached to the N-terminus. In contrast to the AMPs, resistance to lipopeptides is generally rare [13]. As previously described in detail, most native lipopeptides, similarly to the AMPs, act via two major mechanisms: inhibition of the synthesis of cell wall compounds and induction of cell membrane lysis [14].

Attempts have been made to produce synthetic AMPs recruiting all the structural advantages of the native AMPs to build improved antibiotic agents [15, 16]. Ultra-short lipopeptides are amphiphilic molecules mimicking detergents, in which the peptide moiety is hydrophilic and the fatty acid moiety is hydrophobic [17]. It was previously shown that these ultra-short lipopeptides are active against a variety of microorganisms. Like native AMPs, their mode of action involves disturbance of the membrane [11].

In dentistry there are several challenges faced by drug delivery, such as maintenance of drug dosage in the presence of salivary flow and enzymatic action that can cleave certain peptides. Consequently, a sustained release mechanism could allow high concentrations of therapeutic agents for prolonged periods of time.

A combination of the structural and functional properties of peptides with those of synthetic polymers has gained significant interest in material design and application. These smart polymeric systems have several advantages over conventional methods, such as ease of manufacture, and administration, biodegradability, and the ability to alter the release profiles of the incorporated agents [18, 19]. Hybrid molecules of peptides conjugated to polymers can be used for various applications, with the advantage of being resistant to enzymatic cleavage and less cytotoxic to human cells [20].

The present study focused on antibacterial evaluation of lipopeptides as part of a sustained release therapeutic means. The hypothesis was that lipopeptides mixed with biodegradable polymers would maintain an effective concentration and be effective against various oral pathogenic bacteria.

## Materials and Methods

### Test materials

#### Synthetic antimicrobial peptides

Four different ultra-short lipopeptides: C16-KGGK, C16-KKK, C16-KAAK and C16-KLLK, synthesized and purified as previously described [11, 21], were tested.

#### Biodegradable polymer synthesis

Poly (lactic acid co castor oil 30:70) (PLACO) and ricinoleic acid-based poly (ester-anhydride) (P(SA-RA)) were synthesized as previously described [22–26]. In brief, PLACO was synthesized by ring opening polymerization of DL lactide (6g) with a 1% w/w solution of stannus hexanoate as catalyst in castor oil (14g) in a 20 mL ampule. The ampule was heat sealed and kept at 140oC for 48h to form the desired pasty polymer (MW 2300). FTIR and 1H-NMR spectral analysis confirmed the structure and the 3:7 w/w ratio. The poly (ester-anhydride) copolymer of sebacic acid (SA) and ricinoleic acid (RA) at a weight ratio of 3:7 [P (SA-RA) 3:7] was synthesized by transesterification, followed by anhydride melt condensation. In the first step, sebacic acid (SA) is polymerized to PSA with a MW of 20000 or higher by the use of acetic anhydride as activation agent. The formed PSA was reacted with ricinoleic acid (prepared from the hydrolysis of castor oil) at a 3:7 w/w ratio. The formed dimers and trimers of RA-SA or RA-SA-RA were reacted with acetic anhydride to activate the carboxylic acids, followed by polymerization into a polyanhydride at 160oC under a vacuum of 20 mm Hg for 7 hrs. The obtained polymer was pasty at room temperature, with a MW of 13000. FTIR and 1H-NMR spectral analysis confirmed the structure and the 3:7 w/w ratio. The active agent powder was gently mixed with the pasty polymers at room temperature and loaded in a syringe for further experimental use.

#### Formulation of AMP-based biohybrid media

The peptide powders were mixed with the pasty polymer to form a homogeneous paste at a ratio of 100 μg peptide: 100 mg polymer, as previously described [27].

### Bacterial strains, cell lines and growth conditions

#### Preparation of bacterial suspensions

*E*. *faecalis* (ATCC # v583), was cultured overnight in 5 mL brain-heart infusion (BHI) (Difco, Detroit, MI, USA) broth supplemented with 2 mg/mL vancomycin (Sigma-Aldrich), at 37°C under aerobic conditions. *S*. *mutans* (ATCC # 27351) was cultured similarly in BHI broth supplemented with 2.77 μg/mL bacitracin (Sigma-Aldrich) and 5% glucose (Sigma-Aldrich), *A*. *naeslundii* (ATCC # 17233) was cultured in Wilkins-Chalgren anaerobe broth (Oxioid Ltd., Basingstoke, Hampshire, England) supplemented with 2% sucrose under anaerobic conditions. *P*. *gingivalis* (ATCC # 33277) and *F*. *nucleatum* (ATCC # 1594) were cultured in Wilkins-Chalgren broth under anaerobic conditions. The top 4 mL of each bacterial tube were transferred to a fresh test tube and the optical density (OD) was determined according to the specific experiment.

### Antibacterial activity

#### Minimal inhibitory concentration

The antibacterial activity of the lipopeptides was determined by microdilution assay as described before [27, 28]. In brief, the bacterial suspension was diluted and aliquots were added to peptide dilutions in phosphate-buffered-saline (PBS) (Sigma-Aldrich) (in triplicate for each concentration) in wells of a 96-well plate (Nunc 96-well microtiter plates, Roskilde, Denmark). OD (595 nm) was recorded with a microplate reader (VERSAmax tunable microplate reader, Molecular Devices, Sunnyvale, CA, USA) at 37°C for 18–24 hrs. The minimal inhibitory concentration (MIC) was determined as the concentration, which prevented visible growth after 18–24 hrs.

#### Antibacterial activity of sustained release lipopeptides

The antibacterial activity of the formulation was tested for one week by a turbidometric assay as described previously [27]. In brief, a total 10 mg of formulation was placed on the side walls of each of 6 wells in a 96-well microtiter plate and 270 μl of medium were added. Every 24 hrs the medium was collected and transferred to a new set of 6 wells and fresh medium was added to the 6 original wells containing the tested formulation. After one week, a 10 μl volume of *E*. *faecalis* suspension was added to each of the 6 wells and bacterial outgrowth was recorded. The plate was incubated at 37°C in a VERSAmax microplate reader and turbidity (OD_650_ nm) changes were recorded, every 20 min for 18–24 hrs.

### Antibiofilm activity

Antibiofilm activity was tested against 72 hrs biofilms formed in 96-well microtiter plates as previously described [27]. Chlorhexidine (CHX) served as control. Confocal laser scanning microscopy (CLSM) was used to verify the vitality of the bacteria in the different biofilm layers. The bacteria were stained with a live/dead kit (Live/Dead *Bac*Light viability kit, Molecular Probes, OR, USA) as described previously [29].

### Biocompatibility

#### Hemolysis of RBC

The test was performed as described previously [30], in a final volume of 100 μL PBS containing different concentrations of the lipopeptides and 100 μL sheep red blood cells (RBCs) [final concentration 4% (vol/ vol)]. Hemoglobin release was monitored by measuring the absorbance of the supernatant at 540 nm. The controls for 0% hemolysis (blank) consisted of RBCs suspended in PBS, that for 100% hemolysis were RBCs suspended in 1% Triton X-100.

#### Colorimetric XTT assay

Cell viability was tested as previously described [31]. In brief, mouse macrophages RAW-246 were cultured overnight in Dulbecco's Minimum Essential Medium (DMEM, Sigma-Aldrich) supplemented with 10% inactivated fetal calf serum (FCS, Biological Industries, Beit-Ha’emek, Israel), 1% L-glutamine (Biological Industries) and 1% streptomycin (Biological Industries), at 37°C in 5% CO_2_. Each formulation of biodegradable polymer and lipopeptide within plastic inserts (Rosenshein, Israel) that were previously sandblasted, was added to eight wells of a 96-well microtiter plate. Then 200 μL of cell suspension were added to the wells and after 24 hrs the XTT assay (Biological Industries) was initiated by the addition of 50 μL activated XTT solution to each well. The microtiter plate was incubated for 2–4 hrs and then monitored by measuring the absorbance of the supernatant at 450 nm in a VERSAmax microplate reader.

### Bacterial membrane disruption

To evaluate the effect of a lipopeptide on the bacterial cell membrane, cytoplasmic membrane depolarization was measured by the DiOC_2_ (3) assay and fluorescence-activated cell sorting, as described below. The *Bac*Light bacterial membrane potential kit (Molecular Probes, Invitrogen, Eugene. OR, USA) provides a fluorescent membrane potential indicator dye, 3, 30-diethyloxacarbocyanine iodide [DiOC_2_ (3)], along with carbonyl cyanide 3-chlorophenylhydrazone (CCCP). At low concentrations, DiOC_2_ (3) exhibits green fluorescence in all bacterial cells. As it becomes more concentrated in healthy cells that are maintaining a membrane potential, the dye self-associates and the fluorescence emission shifts to red. CCCP is included in the kit for use as a positive control because it is a proton ionophore and it eliminates the bacterial membrane potential. All bacterial suspension samples (1 mL) were incubated in Eppendorf tubes for 1 hr. Then the samples were filtered through a cell strainer 70 μL (SPL life scientific, Korea). A 10 μL volume of CCCP was added to the control group and 10 μL of DiOC_2_ (3) were added to each sample. The samples were kept at room temperature for 30 min before analysis by flow cytometry (BD accuri C6 Flow Cytometer, BD Bioscience, Becton, Dickinson and Company). Whereas the relative red and green fluorescence intensity varies according to cell size and aggregation, the ratio of red to green fluorescence intensity can be used as a size-independent indicator of membrane potential. The data were analyzed with De Novo FCS Express software.

### Sustained release kinetics

The release profile of the lipopeptide from the biodegradable polymer was tested by the fluorescamine assay due to its high sensibility and specificity for primary amino groups, as described previously [32, 33]. In brief, 2 mL of assay solution containing 1800 μL peptide released from the polymer in boric acid buffer (Sigma-Aldrich) together with 200 μL of fluorescamine reagent were introduced into a 12 X 75 mm glass tube. A 200 μL volume of formulation was added to each assay. Fluorescamine (Aldrich Chemical, Milwaukee. WI) was prepared in acetone (Fisher Scientific) to a final concentration of 0.1 mg/mL in a glass screw cap tube. The assay buffer was vortexed until the solutions were completely mixed. Samples were transferred to a polystyrene cuvette that was previously cleaned with nitric acid followed by several rinses with deionized water. Fluorescence was measured at ambient temperature with a Spex 1680 spectrofluorometer (λ_ex_ = 390, λ_em_ = 460) and a time-based scan mode with 2 sec integration time. The measurements were corrected for lamp intensity fluctuations and for the background fluorescence from a solution containing buffer and fluorescamine solution. The final amount of peptide released from the polymer was calculated according to calibration curves made before the experiment.

### Statistical analysis

The data are presented as the mean and standard deviation of a representative experiment performed in triplicate. Multiple comparisons were calculated with Student's t-test. The level of significance was p < 0.01.

## Results

### Antibacterial activity

#### Minimal inhibitory concentration

The MIC for each of the tested lipopeptides against the tested microorganisms are presented in Table 1. The different bacteria showed varying susceptibility to the various lipopeptides. The most potent lipopeptide against *E*. *faecalis* causing complete growth inhibition was KGGK as previously shown [27]. The most effective lipopeptides against *S*. *mutans* were C16-KGGK and C16-KLLK. KKK exhibited antibacterial activity at low concentrations against *F*. *nucleatum*, *P*. *gingivalis* and *A*. *naeslundii*.

**Table 1:** MICs of the AMPs tested [μg/mL].

	Amino acid sequence	*E*. *faecalis*	*S*. *mutans*	*F*. *nucleatum*	*P*. *gingivalis*	*A*. *naeslundii*
**C16-KGGK**	CH_3_(CH_2_)_14_CO-KGGK- NH_2_	4–5	6–12.5	12.5–25	12.5–25	12.5–25
**C16-K****K****K**	CH_3_(CH_2_)_14_CO–KKK- NH_2_	6–12.5	12.5–25	4–5	6–12.5	6.25–12.5
**C16-KA****A****K**	CH_3_(CH_2_)_14_CO—KAAK—NH_2_	12.5–25	>25	12.5–25	>25	>25
**C16-KL****L****K**	CH_3_(CH_2_)_14_CO–KLLK—NH_2_	6.25–12.5	6–12.5	12.5–25	6–12.5	>25

The underlined amino acids are D-enantiomers.

#### Sustained release and antibacterial activity

**Fig 1:**
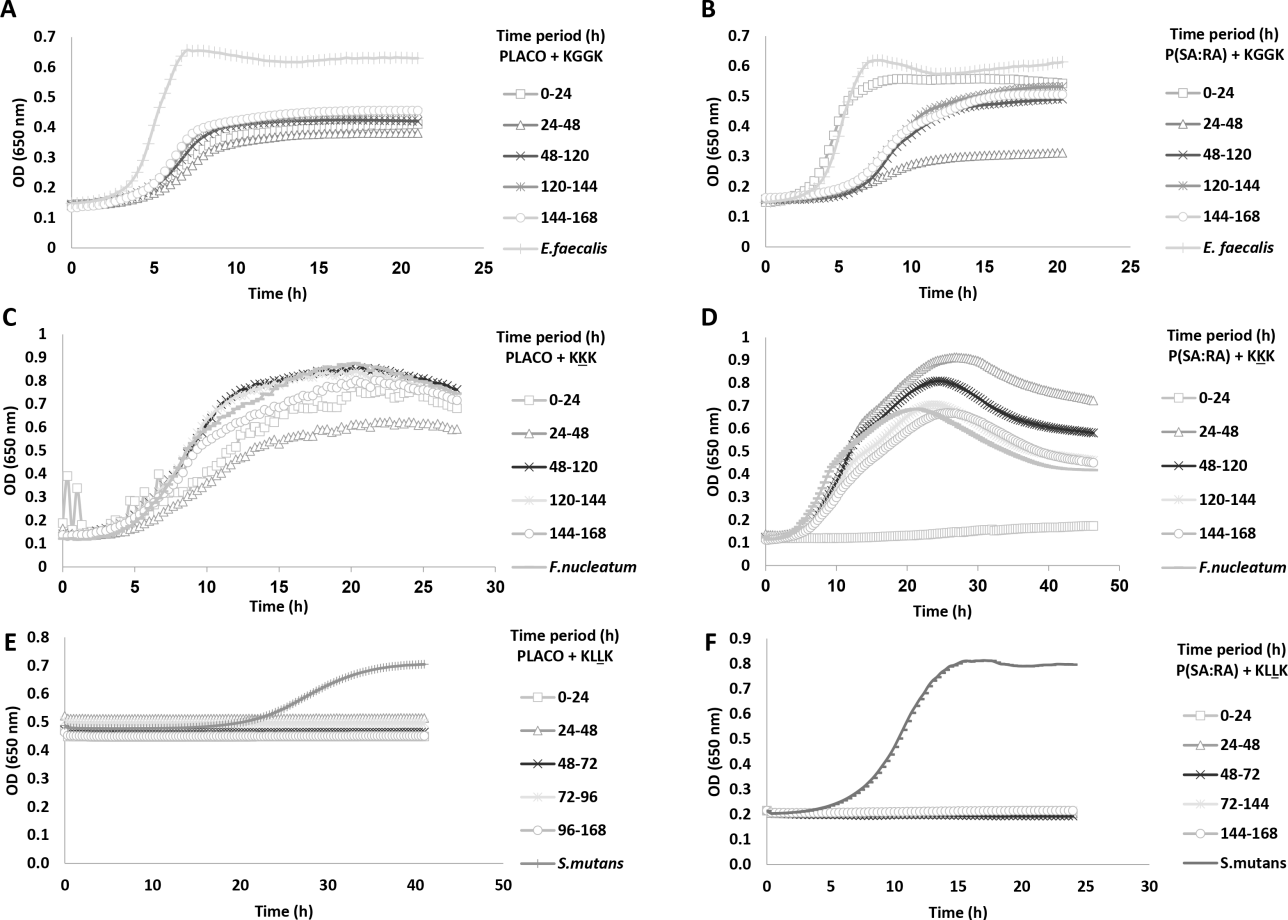
Growth inhibition of bacteria by lipopeptides released from P(SA:RA) or from PLACO. The tested formulation 100 μg peptide + 100 mg polymer, ratio 1:1000 was evaluated for its antibacterial effect every 24 hrs during 1 week. (A, B) KGGK formulations against *E*. *faecalis*. (C, D) C16-KKK formulations against *F*. *nucleatum* and (E, F) KLLK formulations against *S*. *mutans*.

The sustained release effect of the lipopeptides obtained from the biodegradable polymer is shown in **Fig 1**. The antibacterial activity was examined for the most potent lipopeptides against each pathogen, as shown in the MIC experiment. Lower final optical densities and milder growth curve slopes for all the bacteria treated with the formulated peptides were recorded. The most significant antibacterial effect was observed between 24–48 hrs, except for the KKK and P(SA-RA) formulation, where it was evident between 0–24 hrs.

### Antibiofilm effect

**Fig 2:**
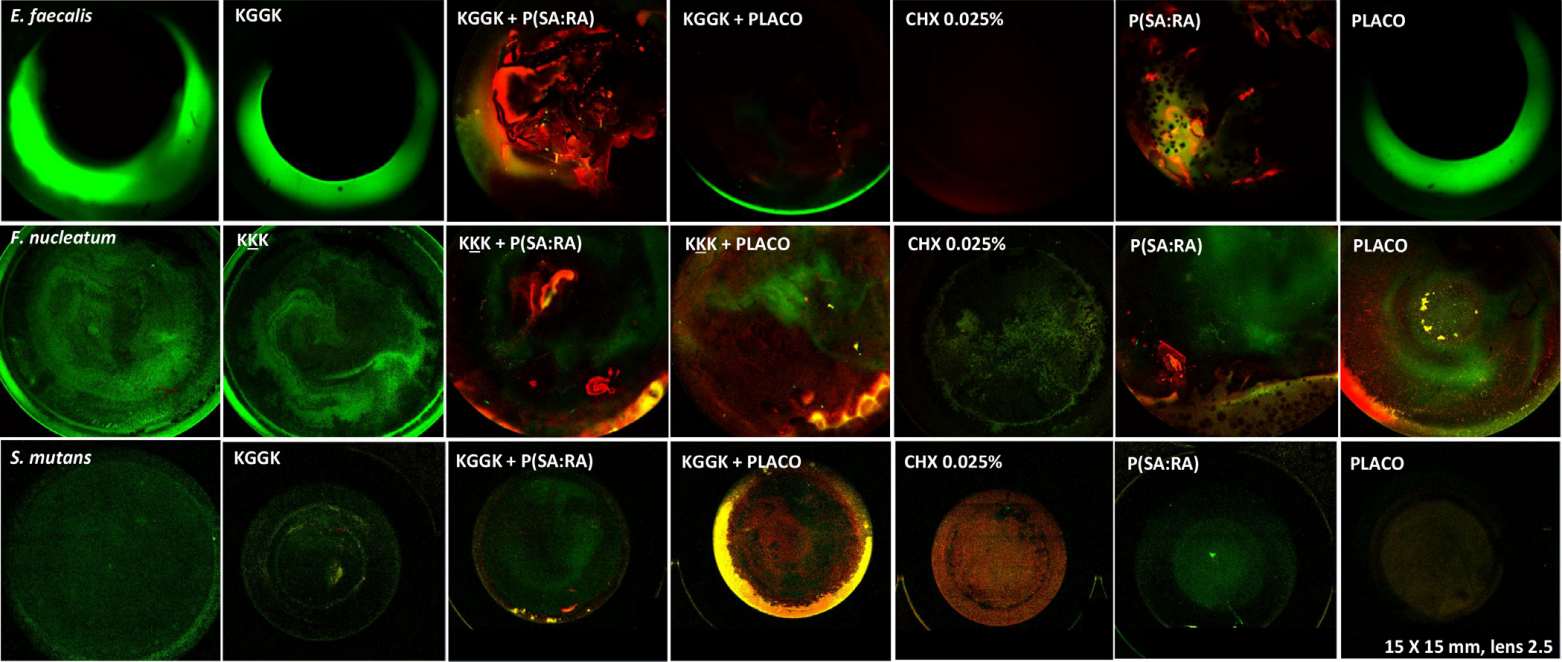
Biofilm growth inhibition by lipopeptide incorporated in biodegradable polymers. The antibiofilm effect was evaluated with the use of a dead/live dying kit against a 72 hr formed biofilm. The live bacteria were stained with a green dye, the dead bacteria were stained with a red dye. Results are shown for *E*. *faecalis*, *F*. *nucleatum* and *S*. *mutans*. The control group included 0.025% CHX.

The anti-biofilm effect against uni-strain biofilms is shown in Fig 2. P(SA:RA) was effective against all the uni-strain biofilms. Formulations containing both the lipopeptides and the biodegradable polymers exhibited a higher antibacterial effect than the non-formulated lipopeptides and CHX.

### Biocompatibility

#### Hemolysis of RBC

**Fig 3:**
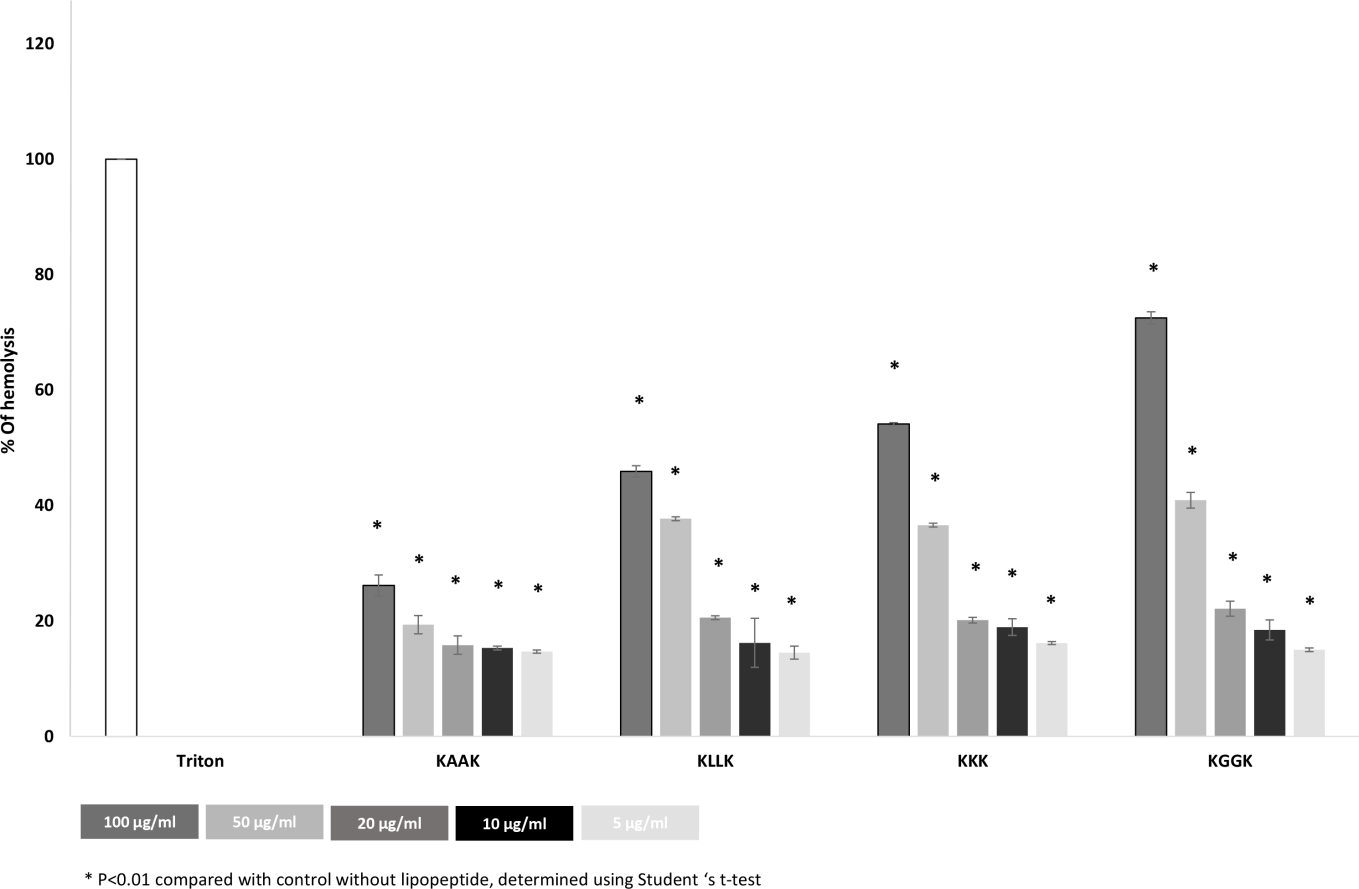
Lipopeptide hemolysis assay. All four lipopeptides: C16-KGGK, C16-KKK, C16-KAAK and C16-KLLK were tested for hemolysis in sheep RBC, at concentrations of 5, 10, 20, 50 and 100 μg/ml. Insignificant hemolysis was detected at the MICs. The control group was considered complete hemolysis.

The results of the hemolysis assay are presented in Fig 3. Lipopeptides C16-KGGK, C16-KKK and C16-KLLK caused high-level hemolysis at the higher concentrations and low-level hemolysis occurred at lower concentrations.

#### Colorimetric XTT assay

**Fig 4:**
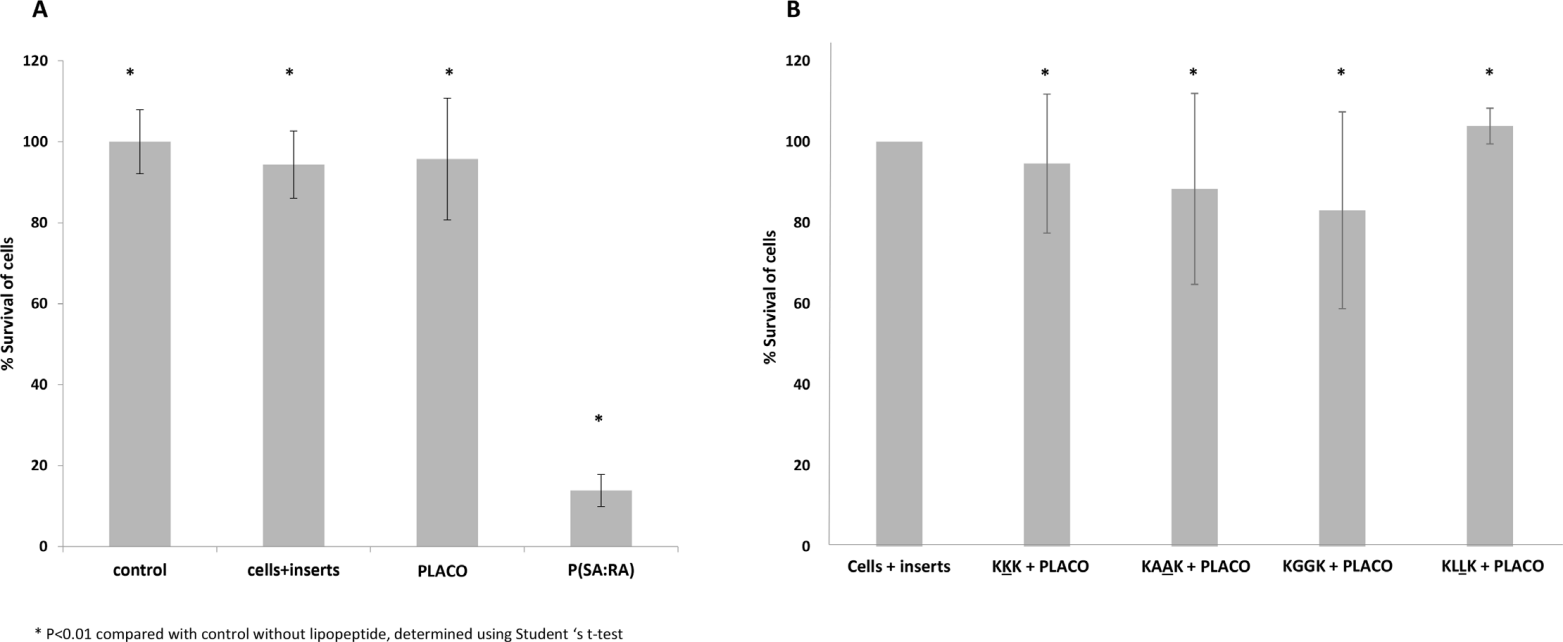
PLACO formulation biocompatibility by the XTT assay. Mice macrophages RAW-246 were cultivated in wells of a 96 well microtiter plate. Each polymer was tested. PLACO exhibited the highest cell survivability (A). Each formulation containing lipopeptide and PLACO polymer was tested. All the formulations showed high cell survivability. C16-KLLK exhibited the highest cell survival vs the C16-KKK, C16-KGGK and C16-KAAK formulations (B).

PLACO and P(SA-RA) were analyzed with the XTT test. The viability of the RAW cells decreased significantly following P (SA-RA) exposure, whereas the PLACO polymer did not affect cell viability compared with that of the control (see Fig 4A). Applications of all four formulations (peptides with PLACO) to the cells resulted in high percentages of cells survival with a minimal decrease in viability vs that of the control. The C16-KKK, C16-KGGK and C16-KAAK formulations led to lower cell survival than the formulation containing C16-KLLK (see Fig 4B).

### Bacterial membrane disruption

**Fig 5:**
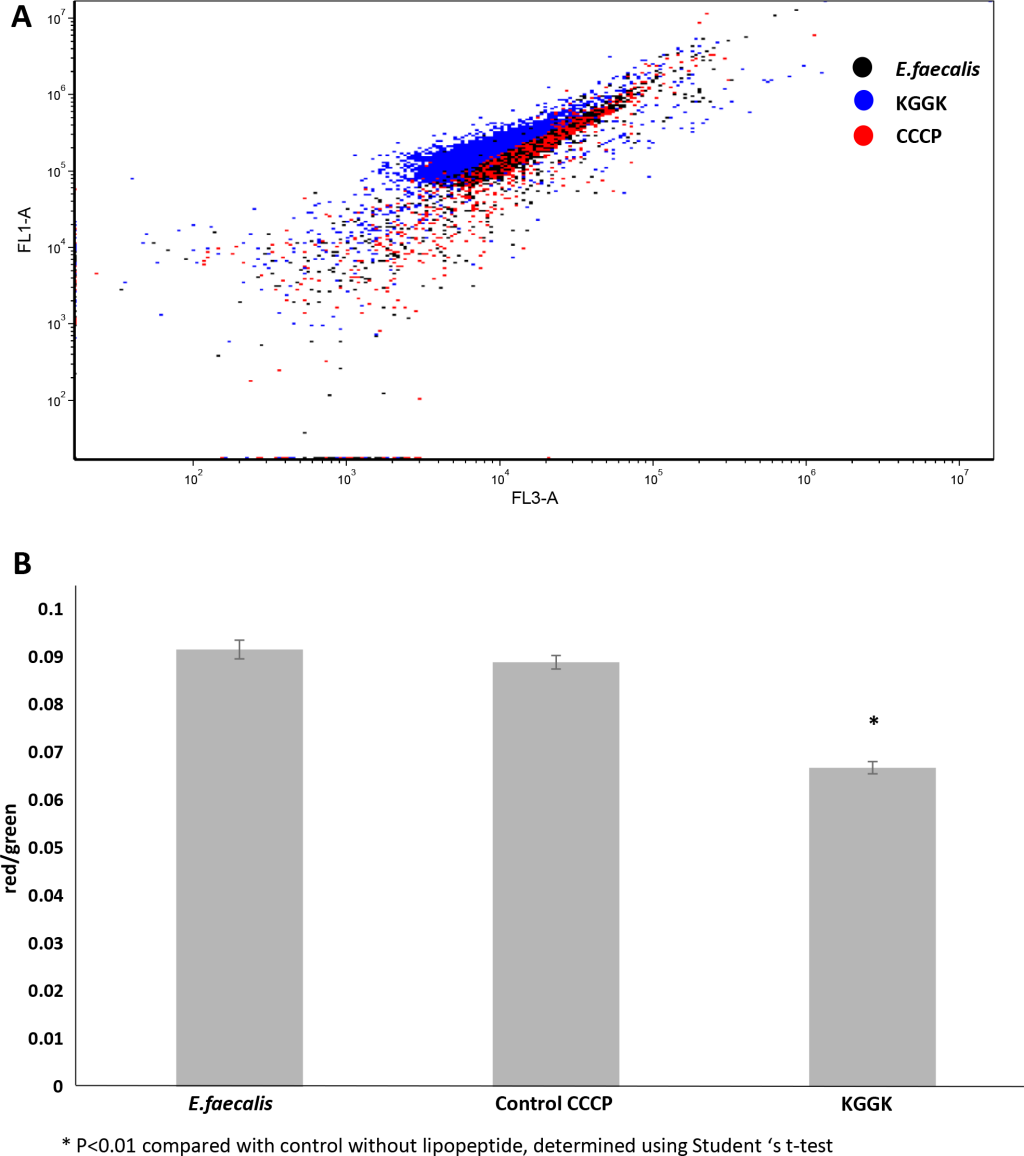
The bacterial membrane is disrupted by lipopeptide after a 1 hr exposure. A representative experiment showing lipopeptide C16-KGGK and its interaction with the *E*. *faecalis* bacterial membrane. Fluorescence-activated cell sorting was used to measure cytoplasmic membrane depolarization and to determine membrane disruption. At high cytoplasmic concentrations the DiOC_2_ (3) self-associates and the green fluorescence emission (FL1-A axis) shifts to red (FL3-A axis). The bacteria were stained with DiOC_2_ (3), exhibiting green fluorescence (FL1-A) with a shift to red emission shift as the dye molecules self-associate at the higher cytosolic concentrations caused by the larger membrane potential (FL3-A). Left shift of the bacteria exposed to KGGK is shown in the dot plot (A). The red/green ratio is lower for the bacteria exposed to C16-KGGK, indicating that the bacterial membrane was disrupted and thus revealing the lipopeptide antibacterial mechanism (B).

Compared with the untreated bacteria, contact with C16-KGGK, increased bacterial staining with DiOC2 seen as a shift to the left (red emission) in the flow cytometry presented in Fig 5A and indicating membrane disruption (see Fig 5A).The ratio between the red and green emission was calculated for each test group (see Fig 5B). The treated bacteria presented lower ratios vs the control, indicating that the bacterial membrane was permeated. CCCP, which was designated to disrupt the cell membrane, did not show depolarization activity against the test bacteria.

### Sustained release kinetics

**Fig 6:**
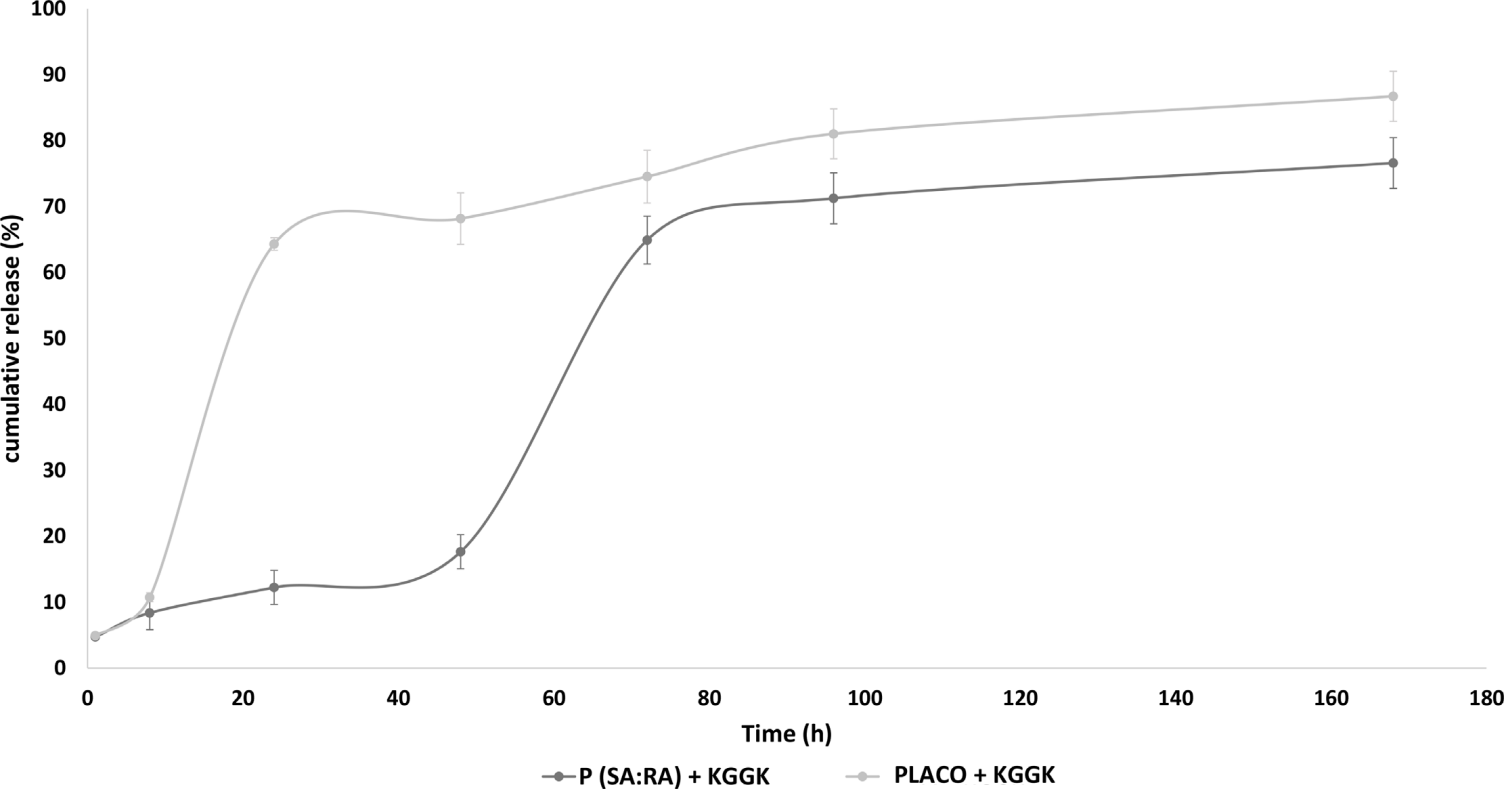
Lipopeptide release profile from biodegradable polymer. A representative release profile of C16-KGGK lipopeptide from P(SA:RA) and PLACO polymers evaluated at 1, 8, 24, 48, 72, 96 and 168 hrs. C16-KGGK release from PLACO peaked after about 72 hrs, whereas the release from P(SA:RA) was continuous throughout the week. The amount of accumulated peptide released from the polymer was calculated according to calibration curves made before the experiment.

The release profile of C16-KGGK lipopeptide from P(SA:RA) and PLACO polymers during one week is shown in Fig 6. The sustained release of C16-KGGK incorporated in PLACO peaked after about 72 hrs, whereas C16-KGGK incorporated in P(SA:RA) was released continuously throughout this period.

## Discussion

**Fig 7:**
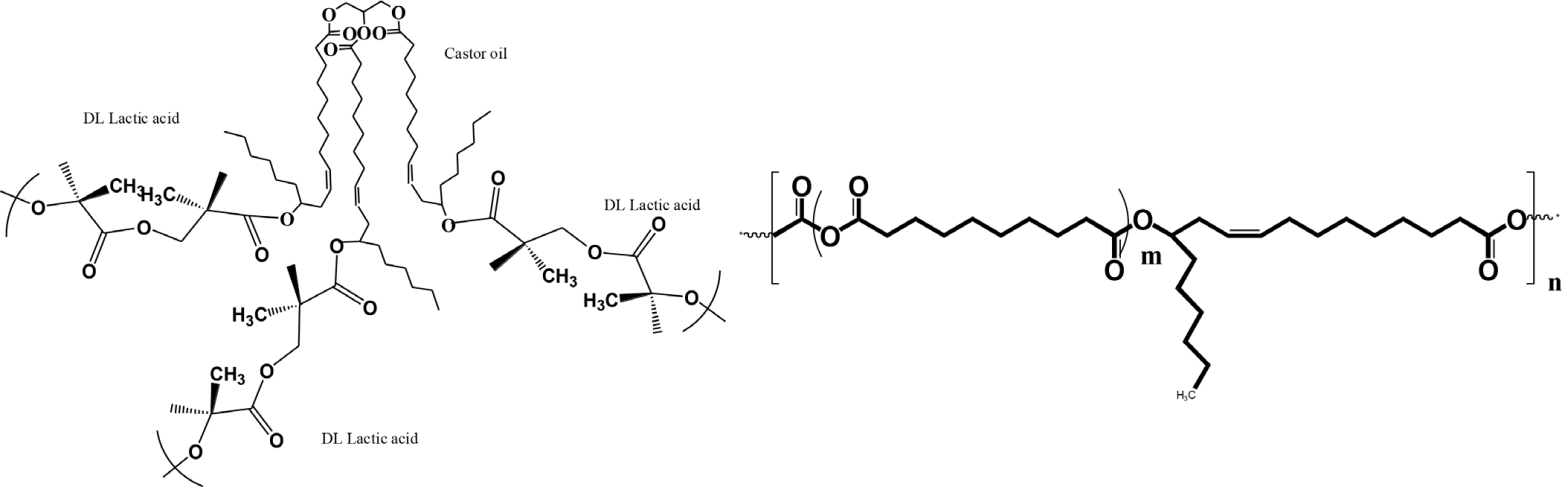
Polymers scheme. Structure of PLACO (left) and P(SA:RA) (right).

The present study focused on antibacterial evaluation of lipopeptides as part of a sustained release therapeutic means. Two biodegradable polymers were: poly (lactic acid co castor oil) (PLACO) and ricinoleic acid-based poly (ester-anhydride) P(SA:RA) (Fig 7). Lipopeptides incorporated in the biodegradable polymers were effective against various oral pathogenic bacteria. The delivery of peptides and proteins by polymeric carriers for extended periods of time has been a challenge because of their instability. Although there are more than 200 peptides and proteins in clinical use and clinical development, there are only a few long-acting drug delivery systems. Luteinizing hormone-releasing hormone (LHRH) and somatostatin delivery systems based on poly(lactic acid), which deliver these agents for months after a single injection, are still the main delivery formulations, developed three decades ago. The challenges of peptide delivery have been reviewed extensively [34, 35]. These novel formulations have broad applications, from cancer immunotherapy to dentistry. There is a wide range of carriers, including lipids, liposomes, nanoparticles and micelles. In the oral cavity, modification of peptides by hydrophobic fatty acid residues or amphiphilic block copolymers has been acknowledged as a useful strategy for protein delivery. In the field of dentistry, polymeric particles and micelles are applicable for binding minerals to the tooth surface, delivering AMPs over a prolonged period of time and thus inhibiting the growth of oral pathogen biofilm in the presence of the saliva pellicle layer [36]. The first polymer for the delivery here was synthesized by ring opening polymerization of DL-lactide onto castor oil that served as co-catalyst for alcohol groups. The second polymer was synthesized by insertion polymerization process that guaranteed alternating ester-anhydride polymer structure. These two polymers are pasty and the incorporation of vulnerable peptides is by gentle mixing without any solvent, heat or sheer stress. Consequently, the activity of these peptidic antimicrobial agents was not affected when incorporated into the delivery system.

The world-wide search for alternative therapeutics against antibiotic-resistant virulent strains of microorganisms, has led to the notion of using cationic AMPs as potential anti-infective compounds [5]. AMPs are known to be the immune system’s first defense line in many organisms [4]. Lipopeptides, which are synthesized by bacteria, share similar characteristics such as cationic charge, amphipathic design and the ability to permeate membranes [11]. Recent studies show that they also have the ability to penetrate the cell, bind to intracellular molecules (DNA, RNA and various proteins) and thereby inhibit the synthesis of cell-walls, nucleic acids and proteins, as well as to inhibit enzymatic activity [37, 38].

To exploit the advantages of AMPs, improved mimetic AMPs were synthesized [15, 16]. Industrial considerations require that the peptides be small and of simple structure. Therefore, considerable research has been devoted to optimize peptide length combined with a simple design, such as: ultra-short lipopeptides. The ultra-short lipopeptides described here, C16-KGGK, C16-KKK, C16-KAAK and C16-KLLK, composed of only four amino acids conjugated to an aliphatic acid chain (16C, palmitate), were synthesized and tested. Studies have revealed that fatty acids are able to compensate for the length of a short peptide chain. Acylation of synthetic or natural AMPs with fatty acids has proved to be a useful approach for improving their antimicrobial and antifungal activity. This effect is due to changes in the overall hydrophobicity of these molecules, which affects their oligomerization, organization in solution and affinity for membranes [39]. The ability of the lipopeptides to oligomerize and turn into aggregates in solution protects them from proteolytic degradation which can affect the half-life of the peptide and its efficacy. As described in detail previously, the in vivo activity of the ultra-short lipopeptides was analyzed in mouse models of fungal infection. Moreover, one of the lipopeptides proved more efficient than the known amphotericin B, at nontoxic doses [14]. The present study focused mainly on the antibacterial effect of these lipopeptides against oral pathogens.

In this investigation, first the MIC for each tested lipopeptide against the bacterial pathogens was determined. Each bacterium was sensitive to a different lipopeptide. Certain lipopeptides, like C16-KGGK, were more efficient against the Gram-positive cocci, whereas other lipopeptides, such as C16-KKK, were more potent against the Gram-negative bacteria. This phenomenon might be explained by differences in membrane structure. It is possible that certain amino acids have a great affinity for specific components, like the lipopolysaccharide (LPS) in the bacterial membrane of Gram-negative bacteria or lipoteichoic acid in Gram-positive bacteria [40]. Next, we focused our evaluation on the most potent lipopeptides. The new biohybrid formulation, polymer incorporating lipopeptide, resulted in an antibacterial effect when tested against bacteria in suspension. This was reflected by both the lower final optical density of the treated bacteria (total growth mass) and the growth rate, measured as the slope compared with that of the untreated bacteria. The most significant antibacterial effect was evident in all experiments after 24–48 hrs of release. It can be assumed that the greatest amount of lipopeptide is released in this time window.

The oral bacterial species tested in this study grow naturally in biofilms within the oral cavity. To determine the antibiofilm effect of the formulations, a live/ dead staining assay was performed on the selected bacteria with the most effective lipopeptides found against them. In all experiments, the biohybrid formulation inhibited biofilm formation, whereas there was no significant effect on the bacteria exposed to the base lipopeptides, indicating that the biofilm prevails over the lipopeptide itself. Consequently, it is likely that the formulation allows continuous sustained release of the antibacterial agent and an anti-biofilm effect. Moreover, it was found that formulations containing this polymer have an added value, as previously shown [27].

As antibacterial agents need to overcome salivary flow and enzymatic cleavage to be sufficiently potent, a sustained release therapeutic can be advantageous in maintaining consistently high peptide levels for local treatment [18, 19]. This must be especially efficient in the intra-oral environment and in the intra root canal dentin tubules where microorganisms such as *E*. *faecalis* are always present and threaten the integrity of teeth and neighboring tissues. Several sustained release delivery devices are already used in dental practice, as the clearance time of most drugs from the oral cavity is rapid and most oral diseases are of a chronic nature [41]. This prompted us to examine four ultra-short lipopeptides, which were incorporated in two selected biodegradable polymers, for sustained release. Peptides and proteins have unique structures that convey their ability to participate in specific biological activities. The fact that these polymers are degraded into natural compounds, renders them environmentally friendly, biocompatible, useful for drug delivery and suitable as implantable devices [42].

The next step was to examine the biocompatibility of the tested lipopeptides as an essential stage of their characterization as therapeutic agents. Two assays were performed. The first (the hemolysis test) tested the lipopeptides and polymers by themselves, and the second (the colorimetric XTT assay) tested the polymers and the formulation containing PLACO and the lipopeptides. Both experiments showed that within the effective concentrations, the lipopeptides alone and the formulations were biocompatible and safe. These results coincide with the results of other, previously performed, hemolysis tests mentioned in the literature [11]. However, in the colorimetric XTT assay P (SA:RA) alone was not found biocompatible and in the hemolysis assay, PLACO showed a high-level of hemolysis. Because PLACO renders the liquid medium cloudy, it can be assumed that it interfered with the absorbance measurements and distorted the experimental results. In this study we used a monocyte cell line to test the cytotoxicity of the tested compounds. In the biocompatibility literature there are many lines used for cytotoxic tests, including monocytes, epithelial cells and fibroblasts. [43]. Monocytes and macrophages are known to play a critical role in the biological response to materials [44], and consequently in the chronic inflammatory response. In comparison with peripheral blood monocytes, the cell lines are more suitable for cytotoxic screening due to their stability and less variation in their response [45]. Nonetheless, before clinical application further *in vitro* and *in vivo* tests are necessary to ensure the safety of their use.

A recent study showed that lipopeptides like C16-KGG, tend to aggregate in solution due to their hydrophobic residues, and alter the intrinsic order of the lipid bilayer upon binding. The cationic lysines make contact with the anionic head-groups of the phosphatidylglycerol lipids, suggesting a model for binding and insertion [46]. In previous studies, scanning electron microscopy revealed bacterial membrane permeation by lipopeptides [11]. In the present investigation, to assess the lipopeptide mode of action and better understand its mechanism, a DiOC_2_ (3) assay was performed. It is known that AMPs operate through membrane disruption, as found here for the tested lipopeptides. Indeed, membrane permeation was detected in this experiment. CCCP did not show depolarization activity against this specific E.faecalis. Previous studies have discussed this issue and it was found that CCCP does not inhibit the efflux pumps of the bacteria, thereby contributing to their resistance [47]. However, more extensive tests should be performed to understand the specific mode of action and the liaison between such lipopeptides and the bacterial membrane.

The addition of fatty acids to biodegradable polymers endows them with flexibility and a low melting point, improves handling and shows efficient degradation and release profiles [42]. All these factors render P(SA:RA) and PLACO suitable candidates for controlled drug delivery. The two biodegradable polymers exhibited different releasing profiles in the fluorescamine assay. Interestingly, in this experiment in the early hours of release, the lipopeptide levels were low. In a previous study accelerated bacterial growth was found at AMP concentrations lower than the MIC [27]. However, it is possible that in the early hours of active lipopeptide release from the polymer, the bacteria can overcome the antibacterial effect if it does not reach the appropriate MIC. Both studies serve to underline the importance of lipopeptide release kinetics. The results seen in Fig 1 can be explained by the release profile of the lipopeptide from the biodegradable polymer. The fluorescamine assay showed the release profile of C16-KGGK from P(SA-RA) and PLACO. After 48 hrs 65% of the lipopeptide was released from PLACO and 15% was released from P(SA-RA). However, these results are not in agreement with the results of the antibacterial activity found in the controlled-release lipopeptides assay in which the most significant antibacterial effect was seen after 24–48 hrs release. This experiment should be performed with other lipopeptides for a better understanding. Although it appears that after one week most of the lipopeptide is released from the polymer; further experiments should be performed for longer periods.

## Conclusions

Antibacterial activity, biocompatibility, mode of action and release profile are four essential parameters for which the biohybrid formulations introduced here were tested. It seems reasonable to assume that the biohybrid formulation containing lipopeptides and biodegradable polymer may be a potential candidate for use in the oral cavity as an antimicrobial means. The advantages of these formulations relate to their reinforced antibacterial activity, ease of manufacture and their ability to cope with a challenging environment like the oral cavity. Nonetheless, as *in vitro* studies have strict limitations, clinical assumptions should be made with maximum precaution.
